# α-Catenin and Piezo1 Mediate Cell Mechanical Communication via Cell Adhesions

**DOI:** 10.3390/biology13050357

**Published:** 2024-05-19

**Authors:** Mingxing Ouyang, Qingyu Zhang, Yiming Zhu, Mingzhi Luo, Bing Bu, Linhong Deng

**Affiliations:** 1 Institute of Biomedical Engineering and Health Sciences, School of Medical and Health Engineering, Changzhou University, Changzhou 213164, Chinaluomingzhi@cczu.edu.cn (M.L.); bubing@cczu.edu.cn (B.B.); 2 School of Pharmacy, Changzhou University, Changzhou 213164, China

**Keywords:** cell mechanical communication, α-Catenin, Piezo1, directional migration, focal adhesion, mechanotransduction

## Abstract

**Simple Summary:**

α-Catenin mediates cell-matrix interactions while Piezo1 at focal adhesions likely mechanosenses traction force through the matrix hydrogel during cell mechanical communication.

**Abstract:**

Cell-to-cell distant mechanical communication has been demonstrated using in vitro and in vivo models. However, the molecular mechanisms underlying long-range cell mechanoresponsive interactions remain to be fully elucidated. This study further examined the roles of α-Catenin and Piezo1 in traction force-induced rapid branch assembly of airway smooth muscle (ASM) cells on a Matrigel hydrogel containing type I collagen. Our findings demonstrated that siRNA-mediated downregulation of α-Catenin or Piezo1 expression or chemical inhibition of Piezo1 activity significantly reduced both directional cell movement and branch assembly. Regarding the role of N-cadherin in regulating branch assembly but not directional migration, our results further confirmed that siRNA-mediated downregulation of α-Catenin expression caused a marked reduction in focal adhesion formation, as assessed by focal Paxillin and Integrin α5 localization. These observations imply that mechanosensitive α-Catenin is involved in both cell–cell and cell-matrix adhesions. Additionally, Piezo1 partially localized in focal adhesions, which was inhibited by siRNA-mediated downregulation of α-Catenin expression. This result provides insights into the Piezo1-mediated mechanosensing of traction force on a hydrogel. Collectively, our findings highlight the significance of α-Catenin in the regulation of cell-matrix interactions and provide a possible interpretation of Piezo1-mediated mechanosensing activity at focal adhesions during cell–cell mechanical communication.

## 1. Introduction

Studies conducted since the 1920s have shown that isolated tissues can establish long-distance connections via fibrous hydrogels. In recent consecutive studies [1], cell–cell mechanical communication has been shown using in vitro experimental models [2], in which mechanical signaling can occur on a large scale and be highly directional. Conversely, chemical signals do not show such precise directionality between individual cells [3,4]. Biomechanics are involved in numerous biological processes. For example, long-range stress transmission across cellular collectives guides endothelial gap junction formation [5]. The fiber matrix is often involved in the perception and response to mechanical force. When cell mechanics induce changes in the location and distribution of type I collagen (COL), mechanical feedback leads to the establishment of a linear tubular model of bistability [1,6]. Studies on collective cell migration and actomyosin in clawed frog cells have shown that contraction of rear cells likely drives collective cell chemotaxis [7,8]. Force promotes the growth of integrin-mediated adhesions and that of mature and newly produced cytoskeletal proteins that enhance adhesion; this process helps cells resist applied forces [9]. Increasing evidence indicates that mechanical signaling plays important roles in cellular communication.

Mechanosensitive molecules mediate mechanical communication between cells. In response to mechanical stimuli, Integrin can be activated to affect cell migration, proliferation, and differentiation [10,11]. During mechanotransduction, Integrin mediates cell-substrate interactions and cellular resistance to mechanical loading [9,12]. Several rapid mechanotransduction responses also rely on mechanogated and mechanosensitive ion channels [13]. For instance, the large transmembrane mechanical force sensory proteins Piezo1 and Piezo2 are mechanosensitive components [14,15]. These ion channels can mediate cell–cell adhesions and the adhesive complexes between cells and the extracellular matrix (ECM).

N-cadherin (N-cad), which maintains the integrity of adherens junctions, mediates the progenitor cell cycle and can restrict the division and differentiation of neural progenitor cells [16]. Piezo channels are functionally connected to the actin cytoskeleton via the cell junctional Cadherin-β-Catenin complex [17]. Various adhesion molecules bind cells to other cells and mediate intercellular and cell-matrix communication. In cells that show increased adhesion and cytoskeletal contractility, cell-matrix interactions are focalized, and the migration mode is mesenchymal migration, which is used by fibroblasts, myoblasts, and numerous types of cancer cells [18,19,20]. α-Catenin is required for acute cadherin-mediated mechanotransduction, while the vinculin-binding site of α-Catenin is required for force-dependent recruitment of actin [21].

To study the mechanisms of distant mechanical cellular interactions, we developed a model based on the rapid assembly of airway smooth muscle (ASM) cells on Matrigel containing 0.5 mg/mL type I collagen (COL) [22]. Our findings showed that cellular mechanical communication is induced by traction force, which is generated by cellular contractions and transmitted through the matrix hydrogel [22]. This traction force is then sensed by Integrin and calcium channels (the critical mechanotransduction components localized at the endoplasmic reticulum and plasma membrane) and N-cadherin, resulting in stable traction force-regulated cell branching connections [22,23]. Recent studies have shown that partial localization of α-Catenin and Piezo1 in focal adhesions can regulate cell spreading and focal dynamics [24,25].

α-Catenin is thought to act mainly as a regulatory cell adhesion protein that can bind to the cytoplasmic domain of cadherin, assemble a protein complex linked to the actin cytoskeleton, and mediate the stability of intercellular adhesions [26]. A previous gene-targeting study showed that α-E-catenin (referred to as α-Catenin in our present study) is required for epithelial cell adhesion formation in mice [27,28]. This study explored the roles of α-Catenin (a mechanosensitive molecule involved in cadherin-mediated adhesion) and Piezo1 (a mechanosensitive ion channel) in long-distance mechanical communication between cells. We investigated whether α-Catenin affects the formation of stable cell–cell and cell-ECM adhesions. Additionally, we examined whether adhesion could be mediated by traction to induce cells to form stable connections to the matrix and whether Piezo1 plays a vital role in this mechanosensing process.

## 2. Materials and Methods

### 2.1. Cell Culture and Reagents

Primary airway smooth muscle (ASM) cells, originated from 6- to 8-week-old Sprague–Dawley female rats [29], were purchased from Beina Biotech. Co. (Beijing, China). All procedures involving animals were approved by the Ethics Committee of Changzhou University on Studies Ethics (Grant No. NSFC 11532003). ASM cells were cultured in low-glucose DMEM (Invitrogen) supplemented with 10% fetal bovine serum (FBS). The cells were cultured in a humidified incubator at 5% CO_2_ and 37 °C. The ASM cells used in the experiments were generally within 10 passages during regular culture.

Matrigel was purchased from BD Biotechnology (San Jose, CA, USA), and type I collagen was obtained from Advanced Biomatrix (San Diego, CA, USA). Low-glucose DMEM, Opti-MEM, FBS, 0.25% trypsin, Lipofectamine 3000, Accutase, and Ctnna1 siRNA were purchased from Thermo Fisher Scientific (Waltham, MA, USA). N-cadherin siRNA (N-cadherin siRNA, #M-091851-01-0005) was purchased from Horizon Discovery (Cambridge, UK), and rat Piezo1 siRNA was from Gene Adv (Suzhou, China). MISSION siRNA universal negative control (#1, SIC001-10 nM), human plasma fibronectin, and DMSO were purchased from Sigma–Aldrich (St. Louis, MO, USA); GsMTx4 was purchased from MedChemExpress (Monmouth Junction, NJ, USA); and GdCl_3_ was purchased from Aladdin (Shanghai, China). A HiScript III 1st Strand cDNA Synthesis Kit (+gDNA wiper) was purchased from Vazyme (Nanjing, China).

The plasmids used in our present study, including those for Integrin α5-EGFP [30], mouse Piezo1-EGFP (Addgene, #80925, Cambridge, MA, USA) [14], and Paxillin-dsRed [31], have been described previously. For inhibitor applications, cells were seeded at appropriate densities in 6-well plates containing hydrogels and allowed to incubate for 30 min; this was followed by the addition of 2 mL of medium supplemented with the appropriate concentration of inhibitor. The concentrations of GsMTx4 and GdCl_3_ were 3 μM [3] and 100 μM [32], respectively.

### 2.2. Preparation of the Hydrogel in a Polydimethylsiloxane (PDMS) Mold

The PDMS mold and cultivation patterns of the cell mesh structure were described in our previous study [22]. A thin layer of PDMS (~500 μm in thickness) was generated as follows. First, the two liquid components included in the Sylgard 184 kit (Dow Corning, Midrand, MI, USA) were mixed thoroughly at a mass ratio of 10:1. Then, this mixture was added to the tablet, air bubbles were removed under vacuum, and the sheet was cured in an oven at 70 °C for 3 h. The PDMS sheet was cut into pieces of appropriate size, and a hole 0.6 cm in diameter was created in the middle using a mechanical puncher. Then, the PDMS was sterilized overnight under a UV lamp in a cell culture hood and attached to the center of a glass-bottom dish (NEST). The liquid mixture was prepared using 100% Matrigel (~10 mg/mL) and pH-neutralized 1 mg/mL COL at a volume ratio of 1:1 on ice. Then, ~50% Matrigel containing 0.5 mg/mL COL was added to the PDMS mold on ice. The assembly was placed into an incubator and allowed to solidify at 37 °C for 30 min. Then, 10–20 μL of cell suspension was added to the top of the hydrogel, and the mold was placed into the incubator for 30 min, followed by the addition of additional culture medium.

### 2.3. Plasmid and siRNA Transfections

ASM cells were cultured to 40–50% confluence in 12-well plates (BIOFIL) and then transfected with 25 nM siRNA using Lipofectamine 3000 (#L3000-008, Thermo Fisher Scientific, Waltham, MA, USA) according to the manufacturer’s protocol. After 8 h, the culture medium was replaced with fresh DMEM supplemented with 10% FBS. The cells were then incubated for an additional 52 h and seeded onto a hydrogel.

For plasmid transfection, ASM cells were cultured to 50% confluence in 12-well plates. Then, 1.5 µg of biosensor DNA, 2.5 µL of P3000, and 2.5 µL of Lipofectamine 3000 were mixed to generate lipid-DNA particles, which were added to the cell culture. After an 8-h incubation, the culture medium was replaced with fresh DMEM supplemented with 10% FBS. The cells were incubated for an additional 30 h and then transferred to the imaging platform.

For siRNA and DNA plasmid cotransfection, siRNA was transfected first, followed by transfection of the DNA plasmid after 24 h. The medium was then changed after 8 h of incubation. Cell imaging was performed approximately 30 h after transfection of the DNA plasmid.

### 2.4. Measurements of siRNA Transfection Efficiency Using qPCR

The downregulation efficiency of ctnna1 siRNA was assessed by measuring the expression of mRNA using quantitative real-time PCR (qPCR). The following qPCR primer sequences were used for rat ctnna1: GTGGGAGGCTCTCCCTAGAA (ctnna1 forward) and CCAGGGTTGTCACCTGTGTA (ctnna1 reverse). The control GAPDH primers used were as follows: AGGTCGGTGTGAACGGATTTG (forward) and GGGGTCGTTGATGGCAACA (reverse) [33]. Primers were synthesized by General Biosystems (chuzhou, China). PowerUp SYBR Green Master Mix (#A25742, Applied Biosystems, Carlsbad, CA, USA) was used according to the manufacturer’s instructions.

### 2.5. Time-Lapse Microscopy and Fluorescence Imaging

The live-cell epi-microscopy system (Zeiss Observer.z1, Oberkochen, BW, DE) was equipped with an X-Y-Z stage for multiple-position imaging, a fine autofocus function for long-duration time-lapse observation, and a constant temperature control chamber (37 °C, 5% CO_2_) to maintain cell viability over time. The imaging interval and duration were 30 min and 16–22 h, respectively. ZEN 2.3 SP1 (blue edition) software was used for time-lapse imaging. Observation and imaging were generally performed using a ×10 objective. Fluorescence images of focal adhesions were acquired using ×100 oil objective via the rhodamine (paxillin) or FITC (Piezo1 or Integrin α5) channel. The parameters of emission filters for rhodamine and FITC were 563 ± 47.5 and 535 ± 12.5 nm, respectively.

### 2.6. Trajectory Analysis of Cell Movement, and Cell Branch Measurement

**Figure 1:**
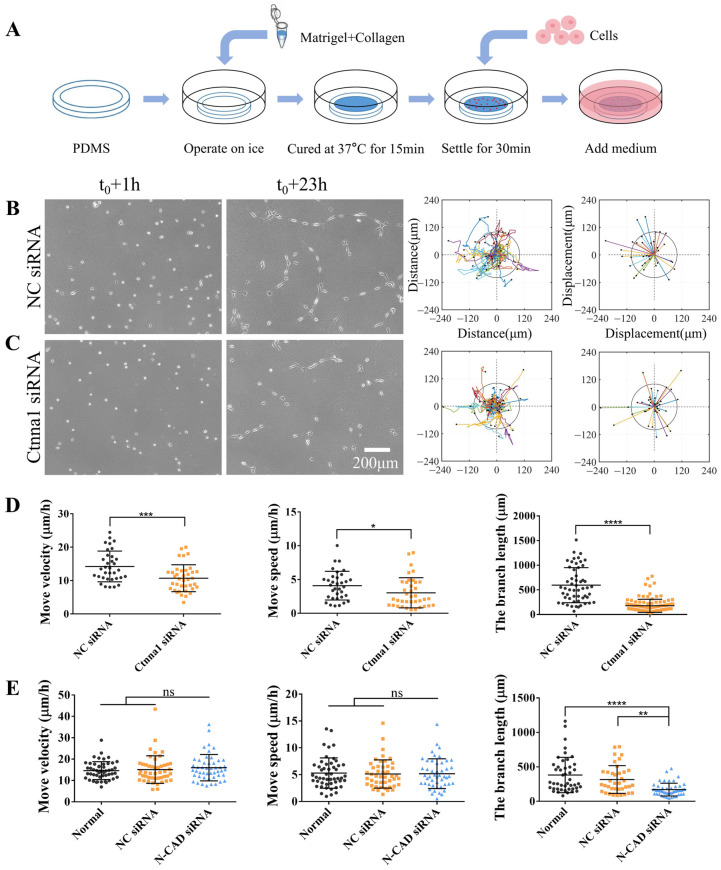
α-Catenin regulates both branch formation and directed migration. ASM cells were transfected with α-Catenin (ctnna1) siRNA or control siRNA. After incubating for 52 h, the cells were seeded onto a hydrogel for time-lapse imaging. (**A**) Experimental setup for cell culture on a hydrogel. (**B**,**C**) Branch assembly of ASM cells transfected with control siRNA (NC) on a hydrogel and migration analysis of trajectory plots (left) and displacement maps (right) (**B**) and of cells transfected with ctnna1 siRNA (**C**). (**D**) Statistical quantification of the ASM cell movement rate and velocity (mean ± S.D., *n* = 34 and 38, respectively) and branch length (*n* = 54 and 118, respectively) under conditions (**B**,**C**). (**E**) Statistical quantification of the movement rate and velocity of ASM cells transfected with control or N-CAD siRNA (mean ± S.D., *n* = 50, 47, and 48, respectively) and branch length (*n* = 45, 38, and 49, respectively). Cell images and migration trajectories are shown in Figure S2. *, **, ***, and **** indicate *p* < 0.05, 0.01, 0.001, and 0.0001, respectively, for significant differences in statistical comparisons; “ns” refers to no significant difference.

**Figure 2:**
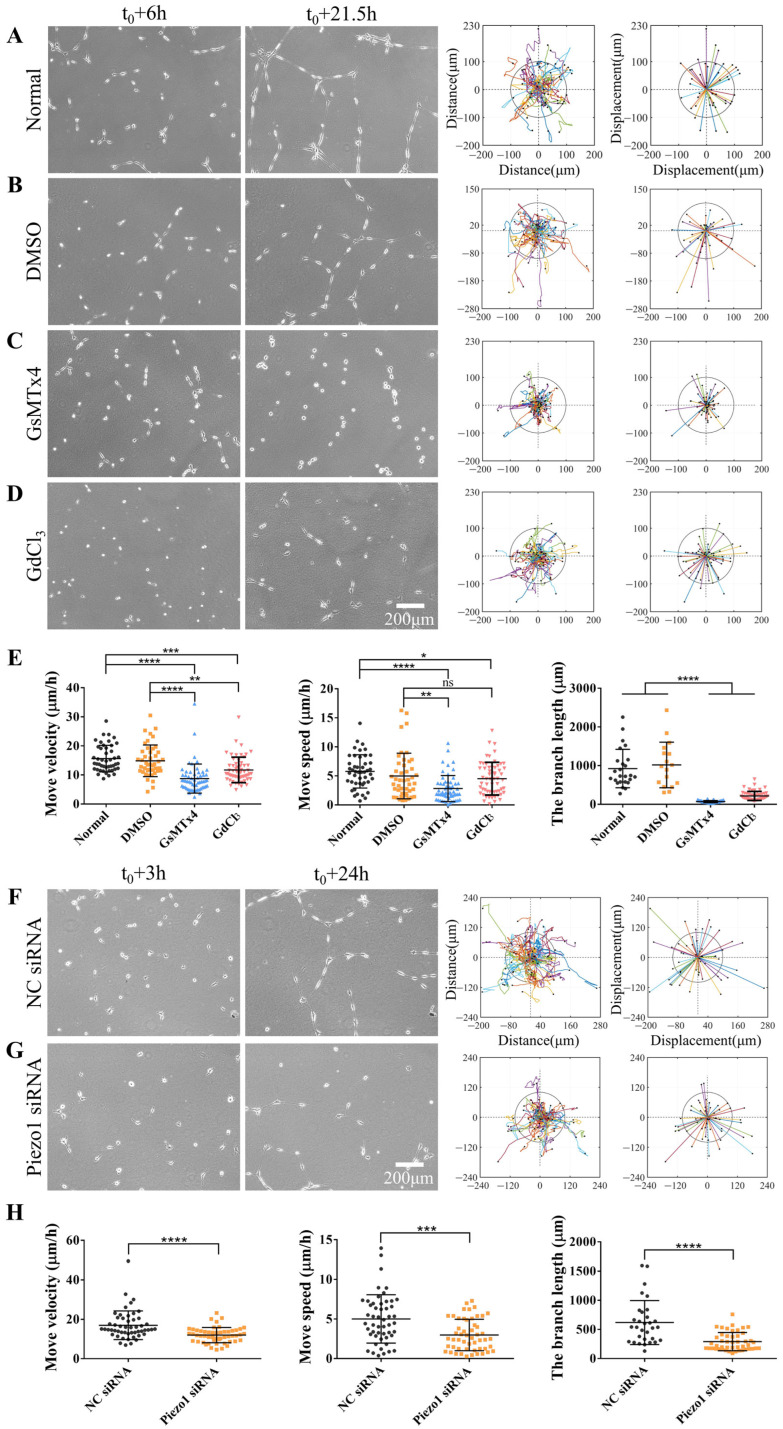
Role of mechanosensitive Piezo1 channels during directed cell migration and self-assembly. Normal ASM cells were inoculated onto a hydrogel with or without an inhibitor in the culture media; DMSO was used for control cells. The cells were allowed to incubate for 6 h, after which time-lapse imaging was performed every 0.5 h for 15.5 h. (**A**–**D**) Cell images at 6 h and 21.5 h. Trajectory analysis plots and displacement maps are shown for the normal state (**A**), control conditions (DMSO) (**B**), treatment with GsMTx4 (3 μM) (**C**), and treatment with GdCl_3_ (100 μM) (**D**). (**E**) Statistical quantification of the ASM cell movement rate and velocity (*n* = 46, 45, 58, and 58, respectively) and branch length (*n* = 25, 17, 49, and 55, respectively) under different conditions (**A**–**D**). (**F,G**) Branch assembly and migration analysis of ASM cells transfected with control (NC) or Piezo1 siRNA on the hydrogel. (**H**) Statistical quantification of the cell movement rate and velocity, and branch length under conditions (**F**,**G**). *, **, ***, and **** indicate *p* < 0.05, 0.01, 0.001, and 0.0001, respectively in statistical comparisons; “ns” refers to no significant difference.

Trajectory analysis of cell movements and cell branch measurements were performed in Figure 1 and Figure 2. To track the trajectories of cell movement, the images acquired under the same field of view were imported into ImageJ in time sequences. Then, the image size was converted to the actual size using the “Set Scale” function. Using the “Manual tracking” function from the plugin list (Plugins –> Tracking –> Manual tracking –> Add track), time-sequence positions (x, y), and moving distances were generated automatically by continuously clicking the target cells through the first to last frame. The digital data files generated in ImageJ 1.53q were collated and imported into MATLAB 2015b software to determine the final rate, velocity, trajectory, and distance traveled (as shown in Figure S1A).

To measure the branch length in ImageJ, the cell at one end of the branch was chosen as the start point, then we drew the line along the branch as long as possible until another end of the branch on the image window was reached by using “Segmented Line”. One branch was defined as cell-to-cell connections continuing from one end to another end. The branch length was measured using the function “Measure” in ImageJ (Figure S1B).

### 2.7. Quantifying the Numbers and Fluorescence Intensity of Cellular Focal Adhesions

**Figure 3:**
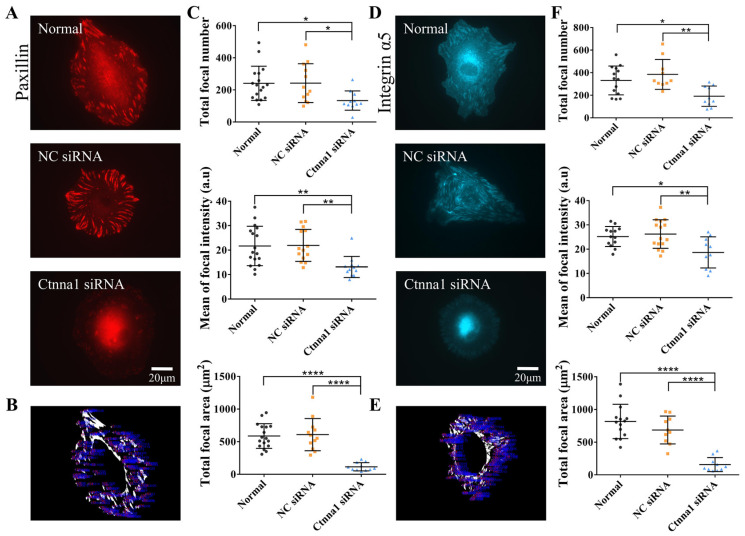
The effect of α-Catenin on the distribution of paxillin or integrin at focal adhesions in ASM cells. (**A**,**D**) Images showing paxillin (**A**) or integrin (**D**) fluorescence in normal ASM cells and in those transfected with control siRNA or ctnna1 siRNA. Fluorescence clusters showing paxillin or integrin α5 expression at focal adhesions. (**B**,**E**) Sample demonstrations for the quantification of the numbers and average fluorescence intensity of focal adhesions based on fluorescent paxillin (**B**) and integrin α5 expression (**E**). The details are described in the Methods section. (**C**,**F**) Statistical analysis of the number of Paxillin-labeled focal adhesions per cell (*n* = 17, 12, and 11, respectively). (**C**) The number of integrin-labeled focal adhesions per cell (*n* = 14, 10, and 10, respectively). (**F**) The average Paxillin or Integrin α5 fluorescence intensity in focal adhesions and the total area of focal adhesions per cell. *, **, and **** indicate *p* < 0.05, 0.01, and 0.0001 in statistical comparisons, respectively; “ns” refers to no significant difference.

**Figure 4:**
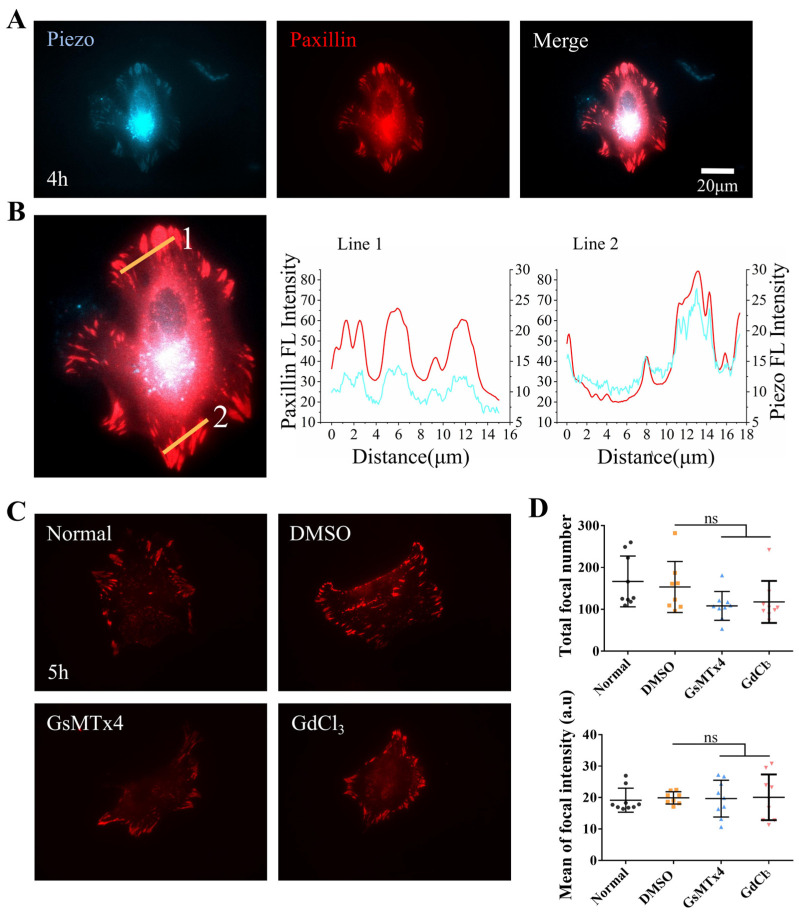
Paxillin and Piezo1 colocalize at cellular focal adhesions. (**A**) Representative images showing colocalization of fluorescent Paxillin and Piezo1 at focal adhesions in ASM cells spread on fibronectin-coated glass for 4 h. Fluorescence images showing Piezo1 expression (left) and Paxillin expression (middle), and merged images showing both Piezo1 and Paxillin expression (right). (**B**) Fluorescence intensities of Piezo1 (blue) and paxillin (red) in regions of cellular focal adhesions in the selected lines. (**C**) Fluorescent paxillin at the focal adhesions of ASM cells in the control group or in the group treated with the inhibitor GsMTx4 or GdCl_3_. (**D**) Statistical analysis of the number of focal adhesions and Paxillin fluorescence intensity (*n* = 9, 8, 9, and 9, respectively) under the conditions described in (**C**). “ns” indicates no significant difference.

**Figure 5:**
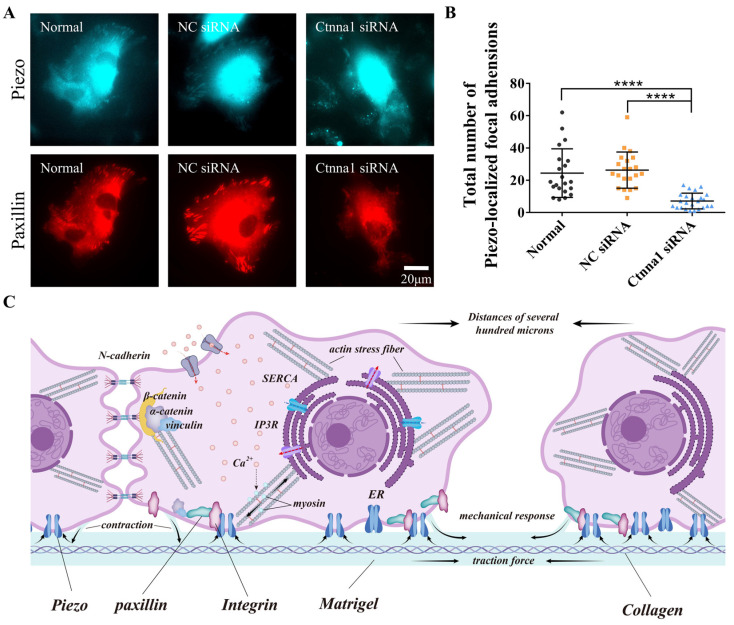
The effect of α-Catenin on Piezo1 focal localization and a hypothetical model of molecular mechanosensation. (**A**,**B**) α-Catenin-mediated regulation of Piezo1 focal targeting. After transfection with control or α-Catenin siRNA, ASM cells were further transfected with fluorescent Piezo1 and paxillin. Images of focal adhesions were acquired (**A**), followed by the counting of Piezo1 focal loci per cell (**B**). Only the cells showing visible focal adhesions were counted. (**C**) Hypothetical model of the roles of α-Catenin and Piezo1 along with actomyosin contraction, Integrin, and calcium channels in cell-to-cell mechanical communication on a hydrogel matrix. Cellular contraction generates traction force, which is transmitted through the hydrogel. α-Catenin regulates both traction force-induced cell–cell adherens junctions and cell-matrix adhesions. Piezo1 partially localizing at focal adhesions together mechanosense the stretch in the matrix for cell–cell distant communication. The arrows indicate the force-pulling directions or targets. **** indicates *p* < 0.0001 in statistical comparison.

Focal adhesion assays were performed in Figure 3, Figure 4 and Figure 5. The nuclear areas showing high fluorescence intensity outside focal adhesions were removed for the analysis of focal adhesion images using ImageJ. The average fluorescence intensity in the focal adhesions was calculated as follows. After “Freehand selections” were selected, the fluorescence-labeled protein region was selected, followed by “Measure” in the Analyze function on the plug-in list. These data were then imported into MATLAB software to ensure that all fluorescence-labeled proteins were counted by adjusting the “threshold” function. The total fluorescence area, average area, and number of focal adhesions were then obtained.

Fluorescence colocalization analysis of two proteins at focal adhesions was performed as follows. Two fluorescence images generated under the same field of view were imported into ImageJ, and the same position was selected to obtain fluorescence data from left to right. The exported data were then summarized using Origin2020. Finally, the fluorescence curves of the two proteins were obtained.

GraphPad and Origin2020 were used for the statistical analysis and generation of the graphs. The values on the graphs represent the mean ± S.D. (standard derivation) from their groups (in scattering dots). *, **, ***, and **** indicate *p* < 0.05, 0.01, 0.001, and 0.0001, respectively, for significant differences between two groups as analyzed using *t*-test. The statistical analysis of multiple groups was conducted according to Analysis of Variance (ANOVA).

## 3. Results

### 3.1. α-Catenin Regulates Cell-Directed Migration and Reticular Structure Assembly in Distant Mechanical Communication

Previous studies have demonstrated the ability of cells to communicate over long distances via force [1,6]. In this study, we expanded on our previously established model (Figure 1A), in which ASM cells use traction forces transmitted through the hydrogel matrix to induce direct migration and rapid branch assembly [22,23]. We transfected ASM cells with α-Catenin siRNA (ctnna1 siRNA) to investigate mechanically induced long-distance cell communication and stable ligation. qPCR analysis confirmed the downregulation of mRNA expression levels by ctnna1 siRNA in ASM cells (Figure S1C). Based on our previous studies, cell directional migration and branch connections were characterized as indices of the efficiency of traction force-induced mechanical communication. Time-lapse imaging was performed every 0.5 h over a period of 22 h to characterize the directional migration of ASM cells on the hydrogel. Our results revealed that ASM cells transfected with α-Catenin siRNA exhibited reduced migration and branch assembly compared with those treated with control siRNA (Figure 1B,C, Supplementary Movie S1). Furthermore, the branch structures formed in α-Catenin siRNA-transfected ASM cells were unstable, resulting in more cell clusters and individual free cells (Figure 1B,C).

In our previous experiments, cell–cell junctional N-cadherin was shown to regulate cell branching assembly but not directed migration [23] (these results are shown in Figure 1E and Supplementary Figure S2). Here, junctional complex-associated α-Catenin influenced both cell migration rates and branching assembly, indicating that junctional complex-associated α-Catenin plays a critical role in both cell–cell distant mechanosensation and stable adherens junctions. Together, these results indicate that α-Catenin, a widely known intercellular adhesion protein, plays a crucial role in the formation of stable branching structures between cells. However, further studies are needed to elucidate the effects of α-Catenin on the rate of cell migration.

### 3.2. Piezo1 Regulates Directed Cell Migration and Branching Assembly

Piezo, an important mechanosensitive ion channel on cellular membranes, is crucial for various mechanotransduction processes [34]. When cells are subjected to mechanical stimuli, Piezo ion channels open to allow cations to enter through the plasma membrane, thereby inducing mechanotransduction [35]. In this study, we examined the role of Piezo1 in cellular mechanical communication and the effects of Piezo1 on the directional migration of ASM cells and their assembly on hydrogels. ASM cells were treated with GsMTx4, a spider venom peptide that inhibits cation infiltration of the Piezo channel family [36,37]. Control cells were maintained under normal culture conditions or treated with DMSO (Figure 2A,B). Our results indicated that the migration rate and velocity of ASM cells treated with GsMTx4 were significantly lower than those of control cells, and inactive single cells were unable to form a stable mesh structure (Figure 2C,E, Movie S2). Similar results were obtained in ASM cells treated with GdCl_3_, a calcium-sensitive agonist (Figure 2D,E, Movie S2). However, the effects of GdCl_3_ were not as robust as those of GsMTx4, suggesting that GsMTx4 may have a more direct effect on the sensing of mechanical forces in ASM cells.

Beside the above chemical inhibitors, we further applied siRNA transfection to more specifically inhibit Piezo1, which showed a more than 60% down-regulation of Piezo1 expression level in ASM cells (Figure S1D). Piezo1 siRNA transfection also markedly inhibited cell migration velocity and speed as well as branch assembly in comparison to control siRNA (Figure 2F–H, Movie S3). Overall, our results indicate that, as a mechanosensitive component of cellular membranes, Piezo1 plays a crucial role in the distant mechanical communication involved in the directional migration and self-assembly of cells.

### 3.3. α-Catenin Influences the Focal Localization of Paxillin and Integrin α5

The above investigation into the roles of α-Catenin in cellular mechanical communication prompted us to ask whether α-Catenin is also potentially involved in cell-matrix adhesions, influencing the rates of cell migration. Hence, ASM cells were transfected with α-Catenin siRNA (ctnna1) for 24 h, followed by cotransfection with either the fluorescent paxillin-dsRed plasmid or the integrin α5-EGFP plasmid, both of which are located at focal adhesions. After plasmid transfection for 24–26 h, the cells were seeded onto 20-mm confocal dishes coated with fibronectin (10 μg/mL), and microscope imaging was conducted after a 4-h rehydration period. The focal scaffold protein paxillin, which is known to localize to discrete sites during cell attachment, interacts with various structures and signaling proteins in response to integrin-mediated cell adhesion [38]. Integrins can induce the assembly of large complexes that bridge the ECM to the intracellular cytoskeleton [39].

In our study, ASM cells expressing integrin α5-EGFP did not exhibit obvious focal adhesions when seeded on a soft hydrogel containing 50% Matrigel and 0.5 mg/mL COL (Figure S3). It is possible that the size of the focal adhesions formed by ASM cells expressing integrin α5-EGFP was outside the resolution limit of our epifluorescence microscopy technique. However, examining the effects of α-Catenin on focal adhesions is still useful for identifying visible focal adhesions in ASM cells seeded on glass surfaces. Our findings revealed that both normal cells and those transfected with control siRNA expressed clearly observable and intact fluorescent paxillin in focal adhesions; however, paxillin expression was markedly reduced in cells transfected with ctnna1 siRNA (Figure 3A). Statistical analysis revealed that the number of focal adhesions, the fluorescence intensity of focal paxillin, and the total paxillin-displayed adhesion area (per cell) were significantly reduced in cells with ctnna1 knockdown (Figure 3B,C). Similar phenomena were detected in ASM cells transfected with fluorescent integrin α5 plasmids, in which siRNA-mediated knockdown of α-Catenin expression markedly reduced the number of focal adhesions, the fluorescence intensity of focal Integrin, and the total area of focal adhesions in the cells (Figure 3D–F). Similar results were obtained when cells were seeded on Matrigel/COL solution-coated glass mimicking the hydrogel substrate (Figure S4A–D). These results suggest that the effects of α-Catenin on the traction force-induced cell migration rate may be linked to the focal localization of paxillin and integrin α5, which play crucial roles in cell adhesion that drives migration.

### 3.4. Partial Localization of Piezo1 at Focal Adhesions

Our results indicated that the expression levels of α-Catenin could affect those of paxillin and integrin α5, resulting in an inability to form stable focal adhesions in ASM cells. Alterations in the cellular perception of mechanical forces via the inhibition of Piezo1 also inhibited cellular motility and connectivity. Therefore, we further examined the relationship between Piezo1 and focal Paxillin in ASM cells.

Fluorescent Piezo1 and paxillin were cotransfected into ASM cells, after which the cells were seeded on fibronectin-coated confocal dishes for imaging. The edges of normal ASM cells showed partial colocalization of fluorescent mPiezo1-EGFP and paxillin-dsRed at focal adhesions at 4 h postseeding (Figure 4A,B). The colocalized fractions were still visible after 25 h, indicating that Piezo1 exhibited focal targeting (Supplementary Figure S5A,B). Subsequently, we treated ASM cells expressing fluorescent paxillin with the Piezo inhibitor GsMTx4 or GdCl_3_. No significant differences in the total number of focal adhesions or the fluorescence intensity of focal paxillin were detected in the boundary regions of treated cells compared with those of controls (Figure 4C,D). Similar results were observed after 25 h (Supplementary Figure S5C,D), indicating that Piezo1-mediated regulation was less involved in the assembly of cellular focal adhesions. Together, these data demonstrate the localization of mechanosensitive Piezo1 at focal adhesions, suggesting that the cell-sensing traction force in the hydrogel matrix was regulated by Piezo1 during cell mechanical communication.

To determine whether α-Catenin also regulates the focal localization of Piezo1, ASM cells transfected with α-Catenin siRNA were further cotransfected with fluorescent paxillin and Piezo1. As shown in Figure 5A,B, Piezo1 localized focally to Paxillin in the control group; however, Piezo1 localization was markedly decreased in the α-Catenin siRNA-transfected group. Our results showed that only a fraction of the cells (~50%) exhibited Piezo1 foci under normal conditions, indicating that the focal localization of Piezo1 was not as stable as that of paxillin or integrin. These results indicate that α-Catenin regulates focal adhesion assembly, which also impacts Piezo1 focal targeting.

## 4. Discussion

Accumulating evidence indicates that cells can communicate over long distances by transmitting mechanical signals through matrix substrates. The specific biological mechanisms underlying this phenomenon remain to be fully elucidated. In our present study, we investigated the mechanism of the α-Catenin-mediated biological response during long-distance cellular communication. Our in vitro model demonstrated that α-Catenin-mediated adhesion between cells and between cells and the matrix can induce rapid and accurate directional migration in cells and promote the formation of a stable tissue mesh structure. Taken together, our recent work has showed actomyosin, Integrin, and calcium channels in mediating cell-cell mechanosensation and N-cadherin in stabilizing the branching connections [22,23], the study conclusions were summarized in Figure 5C. Mechanical forces are essential for the formation of this mesh structure.

ASM cells transfected with control siRNA formed a mesh structure on the hydrogels. However, compared with control cells, ASM cells transfected with ctnna1 siRNA could not form a stable structure and exhibited an inhibited migration rate (Figure 1B–D). These results indicate that α-Catenin promoted both directional migration and mesh structure formation during cell-to-cell distant mechanical communication.

In this study, we have once again underscored the significance of mechanical forces in long-distance intercellular communication. Piezo1, a mechanotransducer, plays a pivotal role in this process. In the Piezo1 ion channel, a specialized transduction structure converts mechanical forces into those used for cation transduction [40]. Therefore, when we inhibited the perception of force by inhibiting the Piezo1 channel, we found that the normal directional migration and self-assembly of cells on the hydrogel were markedly reduced (Figure 2A–H, Movies S2 and S3). In particular, Piezo1 localized to focal adhesions (Figure 4A,B). These results provide mechanistic insights into the role of Piezo1 in mechanosensing the traction force transmitted through the hydrogel matrix. These findings provide evidence that mechanotransduction is imperative for intercellular communication and adhesion.

Paxillin, a multidomain protein, is localized to cell adhesions via its LIM domain. Paxillin can bind to numerous proteins that affect actin cytoskeletal tissue by directly interacting with β-integrin at its tail or with intermediate proteins [41]. Integrins are mechanosensitive receptors on the plasma membrane that facilitate cell-extracellular matrix adhesions [42]. In this study, we investigated the influence of α-Catenin on the focal localization of paxillin and integrin α5 in our in vitro model. Previous research has shown that α-Catenin plays a role in cell adhesion [43]. Our findings demonstrated that knockdown of α-Catenin expression in ASM cells inhibited the focal localization of paxillin and integrin α5, which inhibited directed cellular migration and the formation of branching structures (Figure 3A–F). These findings suggest that paxillin and integrin play essential roles within cells and that the expression of α-Catenin can inhibit cell–cell and cell-stroma adhesions.

Our findings demonstrated that knocking down α-Catenin or inhibiting Piezo1 expression in ASM cells exerted similar inhibitory effects. Therefore, we investigated whether Piezo1-mediated mechanosensation is involved in the formation of focal adhesions via coexpression of Piezo1 with paxillin. Cotransfection of Piezo1 and paxillin plasmids into ASM cells resulted in partial colocalization of Piezo1 and Paxillin (Figure 4A,B), while reducing the expression of α-Catenin via siRNA inhibited the focal targeting of Piezo1 (Figure 5A,B). Taken together, these findings indicate that the inhibition of Piezo1 expression attenuated the directional migration of ASM cells via cell-to-cell mechanical communication (Figure 2A–E). It is reasonable to speculate that the cellular mechanosensing of traction force in the hydrogel matrix is partially due to Piezo1 localization at cellular focal adhesions, although the role of nonfocal Piezo1 has not yet been verified.

## 5. Conclusions

In this work, we showed that α-Catenin is involved in cell adhesion and mechanical sensation in cell mechanical communication. In addition to the connections between N-cadherin and the actin cytoskeleton at adherens junctions, α-Catenin was also functionally associated with focal paxillin and integrin α5, which affected the maturation of cell-matrix adhesions. We also verified the importance of Piezo1 in mechanical transduction and found that Piezo1 was mechanistically involved in cellular mechanosensation during distant mechanical communication. Combined with the results of our recent study showing that the sensing of cell-traction force induces distant mechanical communication [22], our present study provides further important insights into the mechanism of molecular mechanotransduction (Figure 5C).

## Data Availability

Data are contained within the article and Supplementary Materials.

## Supplementary Materials

The following supporting information can be downloaded at: https://www.mdpi.com/article/10.3390/biology13050357/s1, Figure S1: Methods for cell migration and branch length quantifications.; Figure S2. N-cadherin influences cell branching formation, but not directional migration; Figure S3. Integrin α5-EGFP expression in ASM cells seeding on COL hydrogel; Figure S4. The effect of α-Catenin on distribution of paxillin or integrin at focal adhesions on Matrigel/COL solution-coated glass; Figure S5. Measurements of Piezo1 localization at focal adhesions. Movie S1. The time sequences of ASM cells transfected with control siRNA, or ctnna1 siRNA showed cell migration and branching assembly after seeding on the hydrogel matrix. Movie S2. The time sequences of ASM cells under normal condition, or treated with DMSO (control), Piezo inhibitor GsMTx4 or GdCl3 after seeding on the hydrogel matrix. Movie S3. The time sequences of ASM cells transfected with control siRNA, or Piezo1 siRNA showed cell migration and branching assembly after seeding on the hydrogel matrix.
