# Supplementary material for: α-Catenin and Piezo1 Mediate Cell Mechanical Communication via Cell Adhesions
Source: Biology (Basel). 2024 May 19;13(5):357. doi: 10.3390/biology13050357 (PMC11118126; doi:10.3390/biology13050357)
Supplement: Supplementary file 1 [file biology-13-00357-s001.zip › Supporting information_2024.05.pdf]

α-Catenin and Piezo mediate cell mechanical communication via cell adhesions

Mingxing Ouyang [1,†,*], Qingyu Zhang [1,2,†], Yiming Zhu [1,2], Mingzhi Luo [1], Bing Bu [1], Linhong Deng [1,*]

[1]Institute of Biomedical Engineering and Health Sciences, School of Medical and Health Engineering, Changzhou University, Changzhou, Jiangsu Province 213164 China

[2]School of Pharmacy, Changzhou University, Changzhou, Jiangsu Province 213164 China

*Correspondence: mxouyang@cczu.edu.cn (M.O.); dlh@cczu.edu.cn (L.D.)

[†]These authors contributed equally to this work.

The supplementary information contains 5 figures and 3 movies.

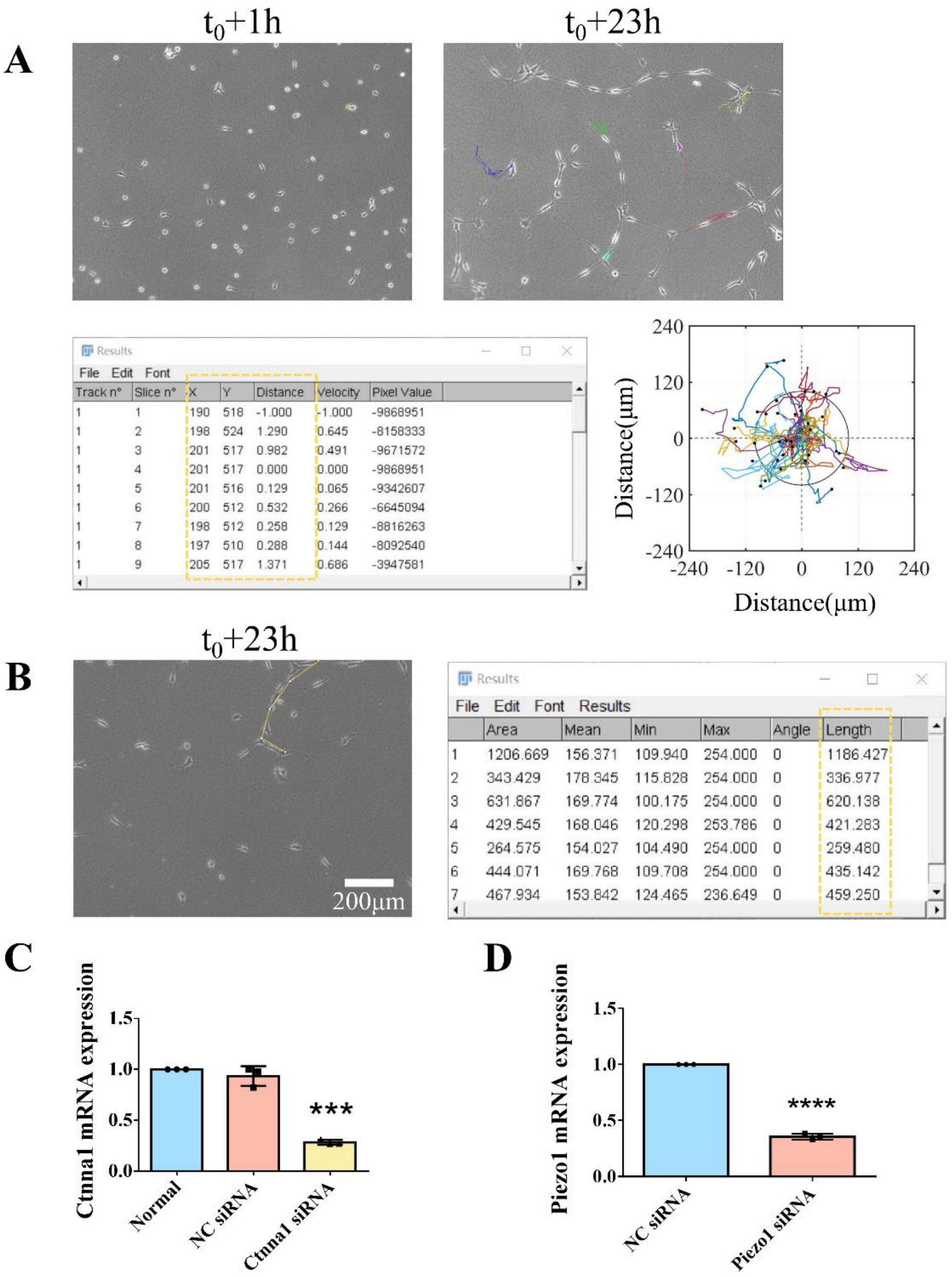

Figure S1. Methods for cell migration and branch length quantifications. (A) Demonstration for trajectory analysis of cell movements, of which details are described in the Methods. **(B)** Branch length measurement through cells under continuous connections within the image window. **(C, D)** Relative mRNA expression levels were determined using quantitative PCR (qPCR) in ASM cells at 72 h, under normal conditions transfected with control siRNA, ctnna1 siRNA (C), or Piezo1 siRNA (D).

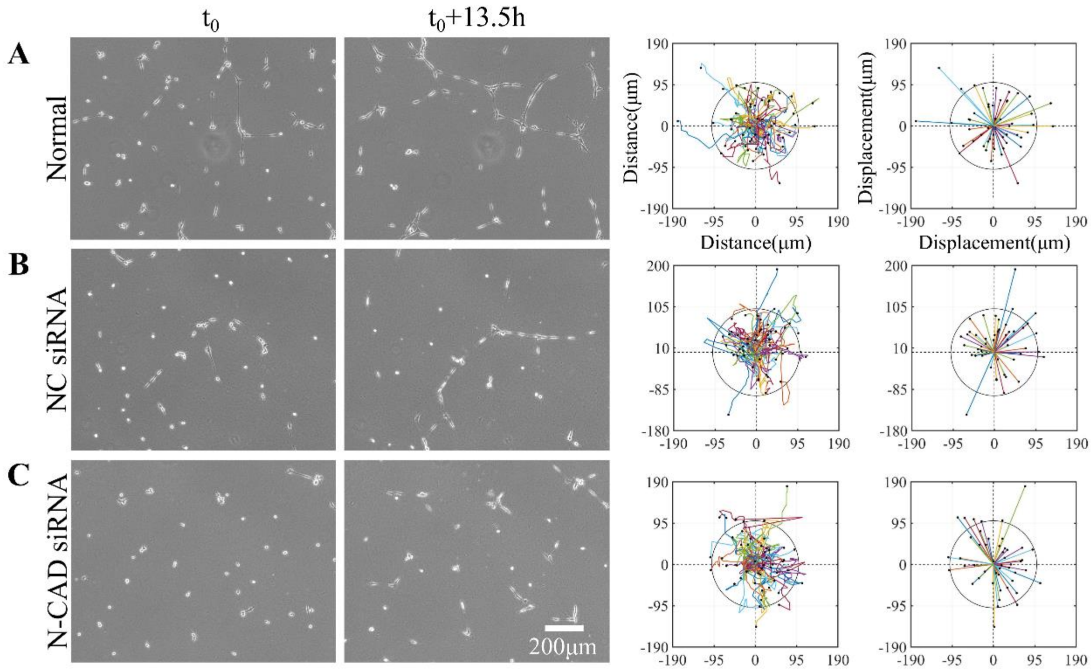

Figure S2. N-cadherin influences cell branching formation, but not directional migration. ASM cells transfected with control or N-CAD siRNA were seeded on Matrigel containing 0.5 mg/mL COL. **(A-C)** Branching assembly and trajectories of cell movements under normal (A), or transfected with control siRNA (B), and N-CAD siRNA (C). The comparisons of corresponding cell move velocity and speed, and branch length are shown in Figure 1E.

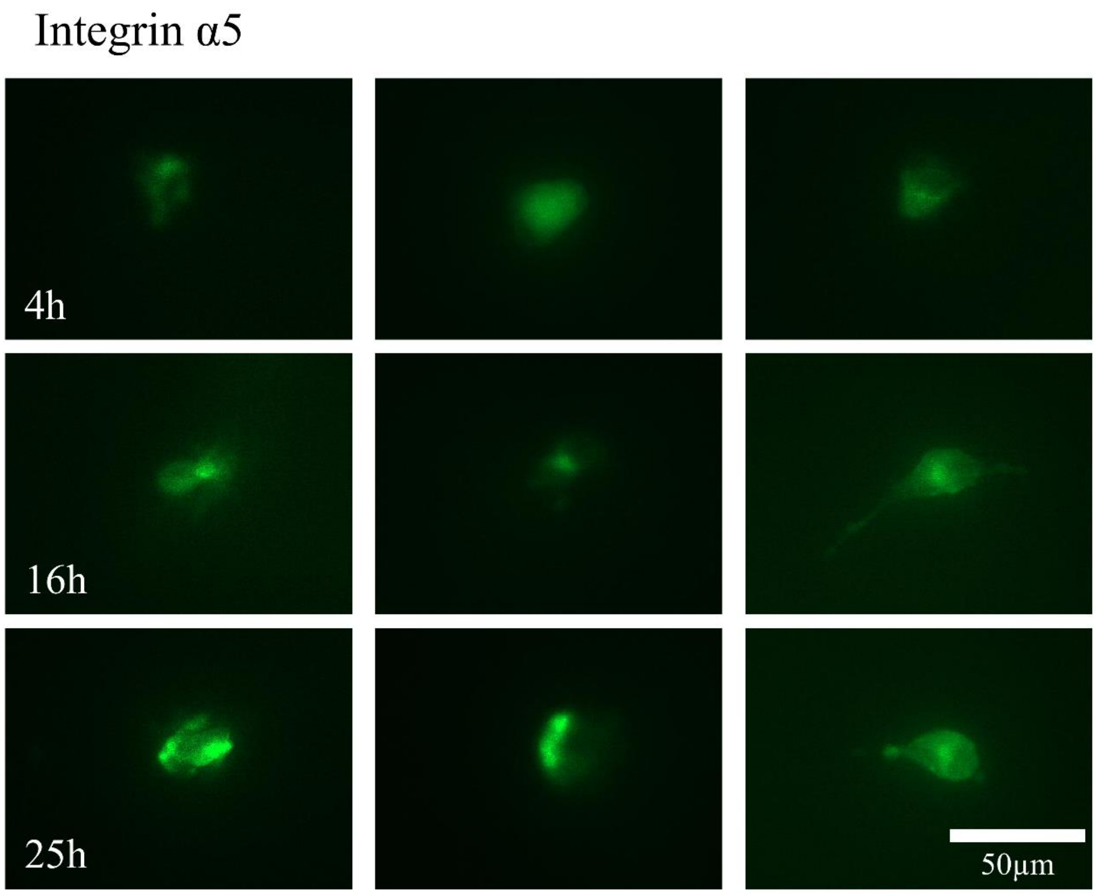

Figure S3. Integrin α5-EGFP expression in ASM cells seeding on COL hydrogel. The cells were transfected with Integrin α5-EGFP and seeded on COL hydrogel for 4, 16, 25 h. Fluorescence images were taken with x40 objective (x100 oil objective not applicable due to the thickness of the 3D hydrogel). Cells were less spread on the soft hydrogel, and very few obvious focal adhesions were visible under the microscope.

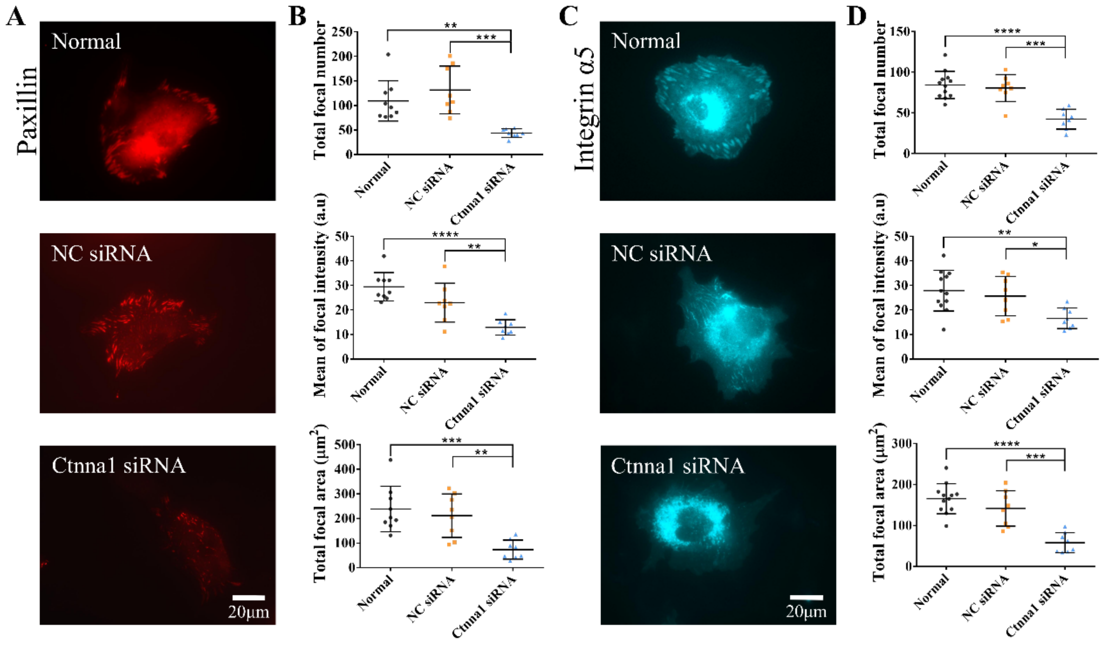

Figure S4. The effect of α-Catenin on distribution of paxillin or integrin at focal adhesions on Matrigel/COL solution-coated glass. The mixture solution of 50% Matrigel and 0.5 mg/ml COL was diluted to 20 μg/ml in protein concentration and added into the glass-bottom dishes for coating overnight. **(A, C)** Paxillin (A) or integrin (C) fluorescence images in normal ASM cells and those transfected with control or ctnna1 siRNA. Fluorescence clusters show paxillin or integrin α5 expression at focal adhesions. **(B, D)** Statistical quantifications for the counted numbers of paxillin-marked focal adhesions per cell (B), or integrin-labeled focal adhesions per cell (D), the averaged paxillin or integrin α5 fluorescence intensity in focal adhesions, and total area of focal adhesions per cell.

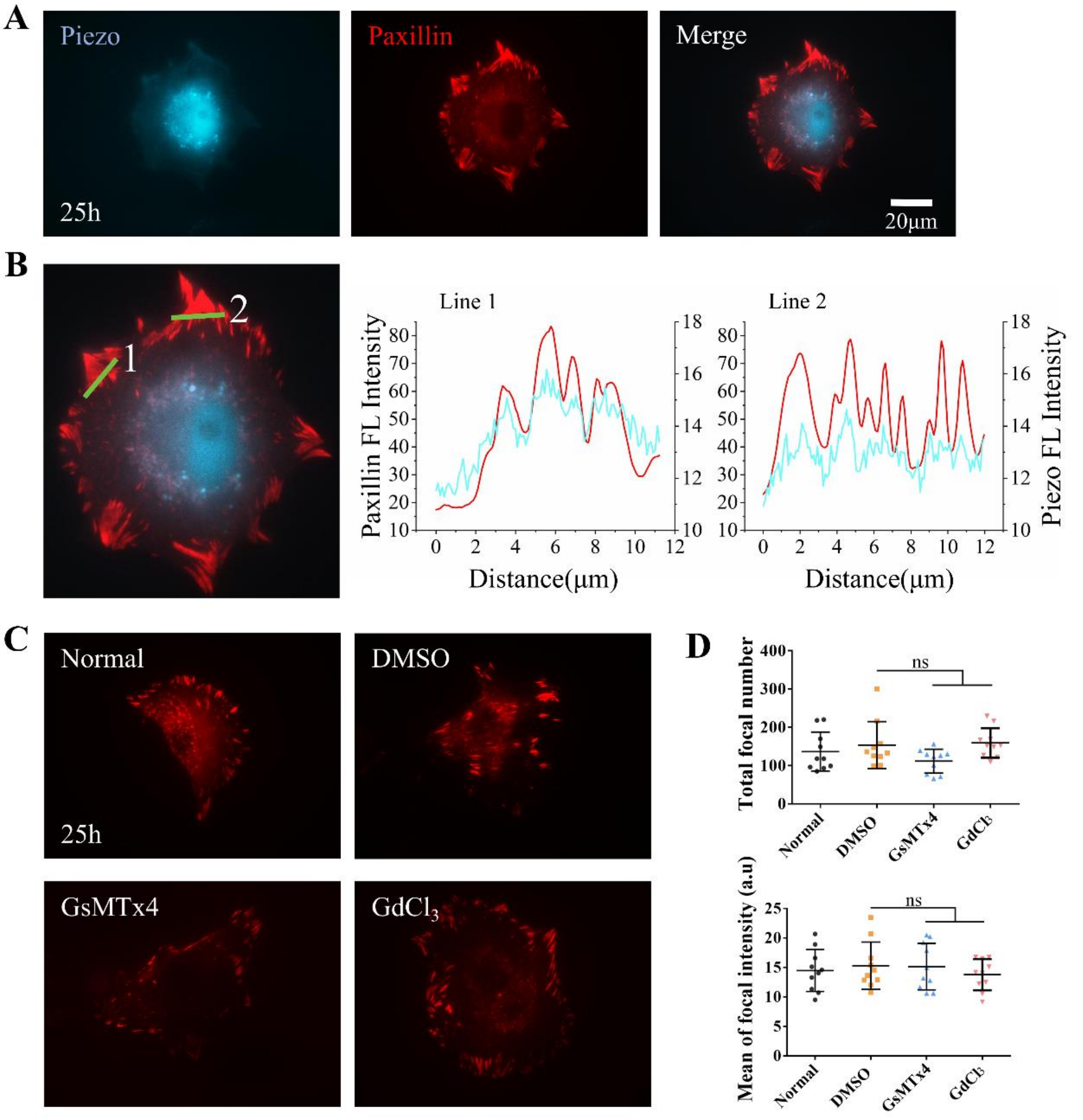

Figure S5. Measurements of Piezo1 localization at focal adhesions. (A) Partial colocalization of Piezo1-EGFP and Paxillin-dsRed transfected in ASM cells on fibronectin-coated glass after 25-h seeding. **(B)** Fluorescence distributions of Piezo1 and Paxillin along the selected two lines to show the local overlays. **(C, D)** Focal adhesion images by Paxillin-dsRed under Piezo inhibitor GsMTx4 and $GdCl_3$ treatments (C), and subsequent quantifications of focal adhesion numbers and average fluorescence intensities at the focal adhesions (D).

Supplementary Movie captions:

Movie S1. The time sequences of ASM cells transfected with control siRNA, or ctnna1 siRNA showed cell migration and branching assembly after seeding on the hydrogel matrix. The time interval = 30 min.

Movie S2. The time sequences of ASM cells under normal condition, or treated with DMSO (control), Piezo inhibitor GsMTx4 or $GdCl_3$ after seeding on the hydrogel matrix. The time interval = 30 min.

Movie S3. The time sequences of ASM cells transfected with control siRNA, or Piezo1 siRNA showed cell migration and branching assembly after seeding on the hydrogel matrix. The time interval = 30 min.
